# Insulin-like growth factor 1 as a key paracrine mediator in mesenchymal stem cell-based intervention for ovarian ageing: a review

**DOI:** 10.1186/s13048-026-02059-0

**Published:** 2026-03-13

**Authors:** Qirui Zhao, Chuan Tian, Jing Zhao, Guifang Chen, Qiang Wang, Junchun Yang, Guangping Ruan, Xilong Zhao

**Affiliations:** 1 Graduate School of Kunming Medical University, Kunming, 650500 China; 2 The Transfer Medicine Key Laboratory of Cell Therapy Technology of Yunan Province, The Integrated Engineering Research Center of Cell Biological Medicine of State and Regions, The Basic Medical Laboratory of the 920th Hospital of Joint Logistics Support Force of PLA, Kunming, Yunnan Province 650032 China; 3 Stem Cell Research Center, School of Medicine, Tongji University, Shanghai, 200331 China; 4 Graduate School of Dali University, Dali, Yunnan 671000 China

**Keywords:** MSCs, IGF-1, Ovarian ageing

## Abstract

**Background:**

Ovarian ageing refers to the progressive functional decline and structural degeneration of the ovary with advancing age, primarily manifested as a reduction in both the quantity and quality of follicles. These changes lead to decreased female fertility, increased miscarriage rates, and elevated risks of age-related diseases. Current treatments cannot fundamentally promote ovarian regeneration. Mesenchymal stem cells (MSCs) possess properties such as self-renewal and paracrine signalling, and their transplantation can restore ovarian function, with the paracrine pathway being a key mechanism. Insulin-like growth factor 1 (IGF-1), a peptide growth factor secreted by MSCs, regulates ovarian cell proliferation, differentiation, and survival through pathways like PI3K/Akt and Ras/Raf/MEK/ERK.

**Objective:**

To summarize and critically evaluate the latest research progress on the role of MSC-secreted IGF-1 in restoring ovarian function.

**Conclusion:**

IGF-1 is an important mediator through which MSCs ameliorate ovarian ageing, improving ovarian tissue structure, hormone secretion, and fertility in preclinical models. However, most evidence remains preclinical, and future work should address the translational challenges, including safety, efficacy, and the precise role of IGF-1 within the complex MSCs paracrine network.

## Introduction

Currently, the postponement of the average age of first childbirth has led to a growing number of women attempting to conceive while experiencing diminished ovarian function. Data indicate that the prevalence of non-iatrogenic Premature Ovarian Insufficiency(POI) has risen from approximately 1% in earlier studies to 3.5% [1]. The decline in egg quality among women of reproductive age, and even the absence of egg development, results in reduced or complete loss of fertility. With advancing age, ovarian function declines because of various factors, including worsening chromosome condensation, telomere shortening, DNA damage and related genetic mutations, oxidative stress, mitochondrial dysfunction, and epigenetic changes [2]. Ageing and degeneration of the ovaries significantly impact women’s physical and psychological well-being. Diminished ovarian function can lead to reduced fertility, increased miscarriage rates, and increased risks of cardiovascular diseases, osteoporosis, depression, and other issues [3, 4]. Therefore, investigating the factors and mechanisms underlying the decline in ovarian ageing can provide theoretical support for enhancing female fertility and improving women’s reproductive health.

Current treatment options for ovarian ageing and related conditions remain limited. While hormone replacement therapy can alleviate menopausal symptoms, it fails to restore fertility and carries risks of thromboembolism and certain cancers with prolonged use [5]. Assisted reproductive technologies such as in vitro fertilization often show reduced success rates in women with diminished ovarian reserve, primarily due to suboptimal oocyte quantity and quality. Other interventions—including antioxidant supplements (e.g., melatonin) and ovarian cryopreservation—have shown promise, though some remain experimental requiring invasive procedures while others demonstrate inconsistent efficacy across populations [6, 7]. There is therefore an urgent need for novel therapeutic strategies to fundamentally restore ovarian function.

MSCs technology, as a promising therapeutic approach, has demonstrated significant efficacy in restoring ovarian function and enhancing fertility in preclinical studies [8, 9]. These cells are widely available from diverse sources including bone marrow, umbilical cord, umbilical cord blood, placenta, menstrual blood, dental pulp, and adipose tissue [10]. MSCs can target damaged tissues, differentiate into multiple cell types, and participate in tissue repair by enhancing endogenous cellular functions, regulating immune responses, and reconstructing the local microenvironment [11]. While paracrine mechanisms are considered crucial for MSC-mediated ovarian repair, the specific key factors and their exact roles remain incompletely elucidated. Among these factors, IGF-1 is frequently associated with promoting cell survival, proliferation, and metabolic regulation. This review critically synthesizes and evaluates existing preclinical and clinical evidence regarding the role of IGF-1 derived from MSCs in mitigating ovarian ageing. We will elaborate on the molecular mechanisms underlying IGF-1’s effects, discuss current challenges in translating these findings into clinical practice, and explore the prospects for developing more effective MSC-based therapies for ovarian ageing.

## Pathophysiological basis of ovarian ageing: multilevel structure and functional decline

### Structural alterations in the ageing ovary

Ovarian ageing is a complex biological process involving changes at multiple levels of ovarian tissue, with a decline in both the quantity and quality of oocytes at its core. At the tissue level, during ovarian ageing, the ovarian vascular network undergoes continuous remodelling, leading to a gradual reduction in vascular density and a consequent decrease in ovarian blood flow [12]. These changes are accompanied by the degeneration of ovarian blood vessels, hyalinization, sclerosis, and stenosis. Reduced blood flow results in insufficient nutrient and oxygen supply to the ovaries, as well as the accumulation of metabolic waste [13]. Additionally, with advancing age, the capacity for vascular regeneration diminishes. The combined effects of various adverse factors in the vascular system contribute to decreases in oocyte quality and developmental potential [14]. Cell count analysis reveals that during ovarian ageing, oocytes gradually deplete, with an increase in atretic follicles and a decrease in healthy follicles in ovarian tissue. These manifestations are also markers of structural and functional degenerative changes in the ovaries [15]. This depletion is also the direct cause of reduced female reproductive capacity. Some studies suggest that in ageing ovaries, there is extensive development of secondary interstitial glands and significant rearrangement of the surface epithelium. Compared with ovaries during the reproductive period, immunohistological changes indicate that interstitial glands become the most abundant tissue during ovarian ageing [16].

### Cellular and molecular hallmarks of ovarian ageing

**Fig. 1:**
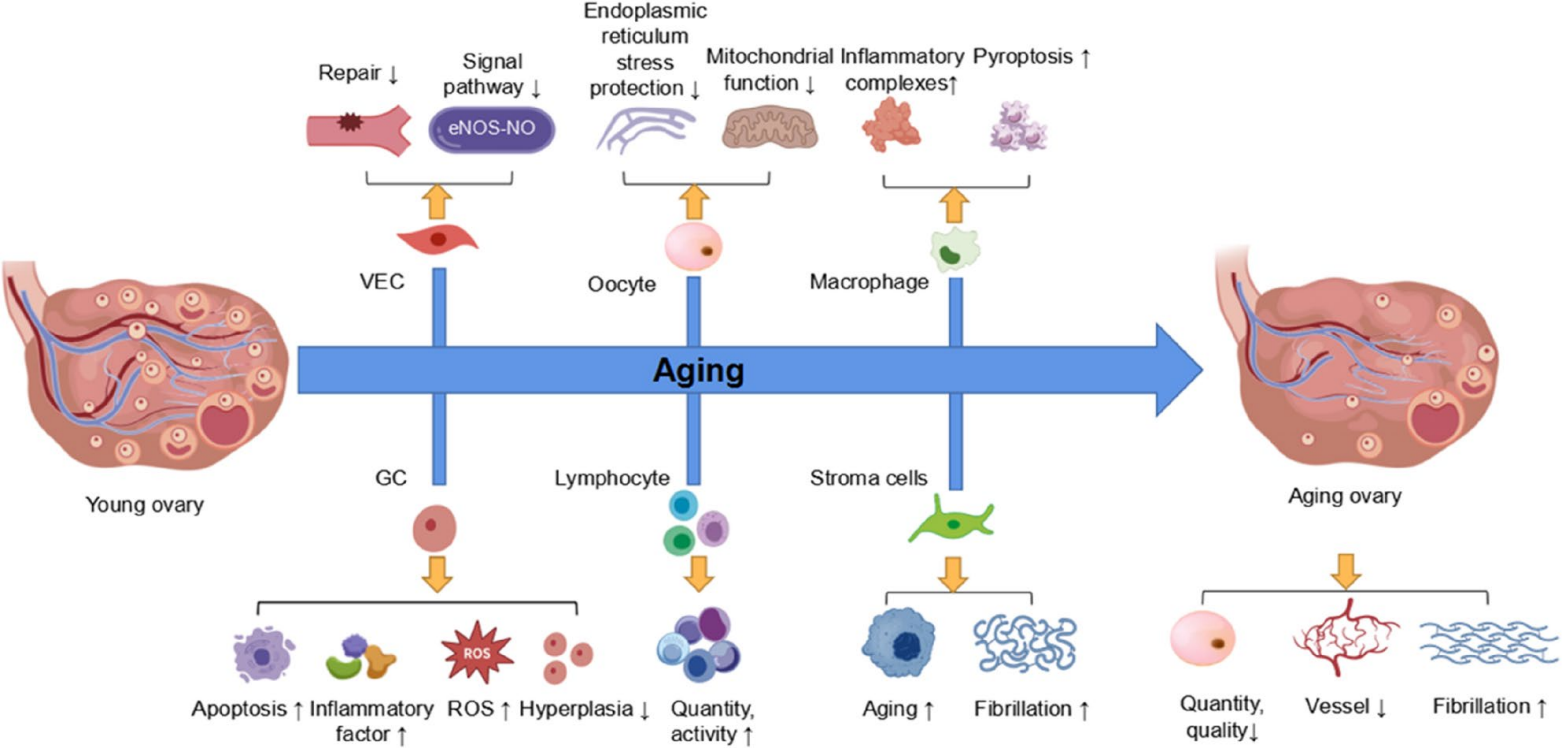
Schematic representation of histopathological and microenvironmental changes in the ageing ovary. GC(Granulosa cell): Increased apoptosis, oxidative stress, immune response activation, reactive oxygen species (ROS) levels, and inflammatory cytokine expression; downregulated mitochondrial function and cell proliferation. VEC(Vascular endothelial cells): Weakened eNOS-NO signaling, impaired vascular repair function. Lymphocytes: Increased number, altered proportion, and enhanced functional activity. Oocytes: Downregulated mitochondrial function and endoplasmic reticulum stress protection. Macrophages: Elevated proportion of pyroptotic macrophages, increased expression of pyroptosis-related enzymes and inflammasome complexes. Stromal cells: Upregulated senescence markers and fibrosis-related expression. Ageing ovary: Reduced oocyte quantity and quality, decreased vascularization, and increased fibrosis.(Created with biogdp.com)

In ageing ovaries, the formation and accumulation of senescent ovarian cells [17] and macrophage-derived multinucleated giant cells [18], as well as increased fibrosis within the ovarian stroma at the molecular level [19], contribute to the damage to follicles and oocytes. Systemic inflammation and elevated levels of proinflammatory cytokines and chemokines also exacerbate this damage [20]. Hormonal monitoring revealed that with increasing age, the secretion of oestrogen and anti-Müllerian hormone (AMH) gradually decreases, whereas the secretion of follicle-stimulating hormone (FSH) increases. Elevated FSH levels lead to reduced telomerase activity and shortened telomere length in GCs [21, 22]. Simultaneously, as ageing progresses, an imbalance in cellular redox homeostasis occurs in ovarian tissue. High concentrations of reactive oxygen species (ROS) in cells cause mitochondrial and nuclear DNA damage, as well as apoptosis. Long-term oxidative stress induced by ROS also contributes to a decrease in the quality of follicles and oocytes [23]. Besides, changes in ovarian microenvironment also have an important effect on ovarian ageing process [24]. Single-cell RNA sequencing analysis of ovarian tissue revealed that the downregulation of genes related to oxidative phosphorylation, endoplasmic reticulum stress protection, and antioxidation in ageing oocytes leads to mitochondrial dysfunction, endoplasmic reticulum stress, and reduced antioxidant capacity [25]. In ageing ovaries, the expression of genes associated with apoptosis, hypoxia adaptation, oxidative stress protection, and immune response activation is increased in GCs [26, 27]. Other changes include an increase in the number of stromal cells [28], accumulation of extracellular matrix [29], elevated fibrotic regulation [30], increased quantity and proportion of immune cells [31], heightened activity [28], and altered intercellular communication patterns [32]. Additionally, eNOS-NO signalling is weakened in ovarian vascular endothelial cells [33], cellular senescence is increased [34], interaction with pericytes is decreased [35], and the activity of vascular endothelial growth factor [36] and hypoxia-inducible factor [37] signalling pathways is reduced, leading to impaired vascular repair function and insufficient angiogenesis around follicles. Accompanied by elevated levels of inflammatory cytokines such as IL-1, TNF-α, and IL-6 [38], these changes trigger chronic ovarian inflammation, oxidative stress, and ovarian fibrosis. The interplay of these multiple factors collectively contributes to the gradual decline in ovarian function. These alterations not only result in reduced female fertility but also may lead to a series of ageing-related diseases. (The changes in the ageing ovary are illustrated in Fig. 1.)

## MSCs: a new strategy for regenerative therapy for restoring ovarian function

### Therapeutic potential and experimental evidence

The beneficial effects of various types of MSCs on ovarian function have been validated in either natural ageing rodents or in chemotherapy-induced ovarian damage models. Studies evaluating the therapeutic potential of bone marrow mesenchymal stem cells (BMSCs) in rat models of chemotherapy-induced ovarian injury revealed that these stem cells could suppress chemotherapy-triggered germ cell apoptosis and DNA damage [39]. When highly purified BMSCs were administered via different routes (intraperitoneal, intraovarian, or tail vein injection) in natural ageing mouse models, the transplanted cells improved ovarian function by regulating mitochondrial function and inhibiting apoptosis. Furthermore, long-term observation and histopathological examination revealed no signs of tumours or abnormal proliferation [40]. MSCs from other sources, such as human placental-derived mesenchymal stem cells (hPD-MSCs) and human adipose tissue-derived mesenchymal stem cells (hAD-MSCs), have also demonstrated significant efficacy in restoring chemotherapy-induced ovarian injury. Transplantation of hPD-MSCs significantly increased serum oestradiol levels, reduced FSH and luteinizing hormone (LH) levels, increased the number of developing follicles, and inhibited the apoptosis of ovarian GCs [41, 42]. hAD-MSCs promoted the recovery of ovarian function through multiple mechanisms, including antiapoptotic effects, hormone secretion regulation, angiogenesis promotion, growth factor secretion, and immunomodulation [43, 44]. When menstrual blood-derived mesenchymal stem cells (MenSCs) treated with 3D alginate hydrogel were incubated with ovaries from aged mice, the follicular status of the ageing ovaries improved. RNA sequencing analysis revealed upregulation and significant enrichment of genes related to mitochondrial pathways. Treatment with MenSCs increased the expression of genes associated with mitochondrial pathways and promoted the restoration of ovarian function in aged mice [45]. These studies not only confirm the effectiveness of multisource MSCs in ameliorating ovarian ageing but also reveal various mechanisms involved in mitigating ovarian ageing, providing potential targets for clinical drug development. It is important to note that the cited studies utilize two main types of preclinical models: natural ageing models, which recapitulate the gradual, multifactorial decline of ovarian function with time; and chemotherapy-induced injury models, which represent an acute, toxic insult leading to premature ovarian insufficiency. While MSCs shows beneficial effects in both, the initiating insults and some downstream pathological features differ, which should be considered when interpreting the results.

### Clinical research progress and challenges

In clinical practice, research on the use of MSCs in the field of ovarian function restoration is still in its preliminary stages, with a limited number of related clinical trials. However, existing studies have yielded some positive outcomes. For example, one study transplanted a collagen scaffold containing human umbilical cord mesenchymal stem cells (hUC-MSCs) into patients with premature ovarian insufficiency under ultrasound guidance. The results revealed a significant decrease in FSH levels, a notable increase in the oestradiol levels, and an increase in the ovarian volume. Ultrasound examinations also revealed improved ovarian blood flow, and some patients achieved natural pregnancy after transplantation [46]. Another experiment involving intraovarian injection of BMSCs demonstrated an improvement in clinical manifestations related to ovarian failure, with the procedure deemed safe and no adverse events observed [47]. Additionally, intraovarian transplantation of autologous hAD-MSCs and autologous BMSCs has been proven to be safe and feasible. Experimental results indicated that stem cell transplantation optimized the mobilization and growth of existing follicles, improved follicle and oocyte counts, and led to the restoration of menstruation and hormone levels in some patients after transplantation [48, 49]. Preliminary studies on peripheral blood-derived stem cells also suggested a significant improvement in hormone secretion in patients with ovarian failure, promoting the resumption of menstruation and pregnancy [50]. In studies on allogeneic transplantation of hUC-MSCs for treating premature ovarian insufficiency, no serious adverse events were observed. The results showed that transplantation improved ovarian function, promoted follicle development, and facilitated the formation of mature follicles. Some patients successfully conceived and delivered healthy infants after treatment [51]. In other studies, allogeneic amniotic epithelial cells transplanted via the ovarian artery significantly improved endometrial thickness, ovarian size, and hormone levels. Some patients resumed menstruation, and menopausal symptoms were alleviated [52]. These findings suggest that MSC transplantation is beneficial for patients with a diminished ovarian reserve, providing preliminary clinical evidence for its application. However, owing to the limited sample size, further large-scale clinical studies are needed to validate its long-term efficacy and safety. Although clinical research still requires further refinement, studies on various types of MSCs for restoring ovarian function have shown significant results in animal experiments [53], providing important scientific evidence for the clinical application of MSCs. The therapeutic effects of MSCs are achieved not only through cell replacement or differentiation but also through their paracrine effects, which play a key role in ovarian function recovery. MSCs secrete various growth factors and cytokines that promote angiogenesis, inhibit inflammatory responses, regulate the immune microenvironment, and improve ovarian function through mechanisms such as autophagy and endocrine regulation [54]. The discovery of these multiple mechanisms suggests that MSC therapy is a potential novel treatment for ovarian ageing. However, transitioning from basic experiments to feasible clinical treatment plans requires more clinically relevant research to verify the actual efficacy and safety of MSC therapy and its related mechanisms in humans [55].

### Challenges in translational application and future perspectives

Despite encouraging preclinical and preliminary clinical results, the translation of MSCs therapy into a routine clinical treatment for ovarian ageing faces several significant challenges. A paramount concern is the potential risk of tumorigenicity. As highly proliferative cells, MSCs carry a theoretical tumorigenic risk during in vitro manipulation and transplantation, necessitating long-term monitoring in human trials. Furthermore, while the immunogenicity of allogeneic MSCs is generally considered low, it still requires careful vigilance, as repeated administrations or specific host conditions may potentially elicit immune responses. Another major challenge is the insufficiency of long-term post-treatment data. Current clinical evidence is primarily based on small-scale, non-randomized studies with limited follow-up durations. Among completed clinical trials on MSCs therapy for premature ovarian insufficiency registered on ClinicalTrials.gov, the currently available publicly reported information indicates that the longest follow-up period is 128 weeks (NCT02372474). These data reflect limited long-term monitoring to date. Data concerning long-term efficacy—such as sustained improvement in hormone levels and live birth rates several years after treatment—as well as long-term safety remain scarce. Conducting larger, randomized, placebo-controlled trials with extended follow-up periods is therefore crucial. Regarding regulatory and manufacturing hurdles, standardizing MSCs sources, isolation, expansion, quality control, and storage is critical for ensuring batch-to-batch consistency and therapeutic reproducibility. However, this remains a significant challenge within the field.

## IGF-1 is a key mediator of the paracrine improvement of ovarian ageing in MSCs

### Evidence and effects of paracrine IGF-1 in MSCs

Multiple studies have suggested that the improvement in ovarian function following MSC transplantation is largely attributed to paracrine mechanisms [15, 56, 57]. After transplantation, MSCs secrete various cytokines, including IGF-1. Through direct or indirect actions, these cytokines promote the secretion of functionally active mediators in adjacent ovarian cells, reduce tissue damage, inhibit apoptosis and fibrosis, promote angiogenesis, and modulate immune responses, thereby repairing ovarian tissue damage and improving ovarian function [44, 58]. Related research has indicated that IGF-1 is an effective stimulator of cell proliferation and differentiation, regulating GC apoptosis and steroidogenesis during follicular development. IGF-1 not only stimulates the production of vascular endothelial growth factor (VEGF) and progesterone in GCs but also synergizes with gonadotropins to maintain the secretory activity of GCs [59]. One study found that extracellular vesicles derived from hUC-MSCs carrying IGF-1 inhibited cyclophosphamide-induced excessive autophagy in GCs by activating the Nrf2/HO-1 signalling pathway, thereby ameliorating cyclophosphamide-induced ovarian damage in mice [60]. A metabolic assessment of aged female rats after the injection of hPD-MSCs revealed weight loss posttreatment. Serum levels of IGF-1 were significantly elevated, and the levels of 3-hydroxybutyrate, glycocholic acid, and taurine—associated with extended healthspan—increased after injection [61]. In a study in which naturally aged rats were used as a perimenopausal model, neonatal hUC-MSCs were transplanted into the treatment group via tail vein injection and promoted ovarian expression of hepatocyte growth factor (HGF), VEGF, and IGF-1 [15]. In another study in which human amniotic-derived MSCs were used to alleviate cyclophosphamide-induced ovarian damage, these cells secreted fibroblast growth factor 2, IGF-1, HGF, and VEGF after transplantation. These growth factors improved the local ovarian microenvironment, leading to reduced Bax expression and increased Bcl-2 expression in ovarian cells, indicating that MSC transplantation may improve ovarian function by inhibiting ovarian GC apoptosis, promoting ovarian angiogenesis, and regulating follicular development [62]. These studies suggest that the paracrine secretion of IGF-1 contributes significantly to the ability of MSCs to alleviate ovarian ageing. The experimental evidence supporting the pivotal role of MSC-secreted IGF-1 and other paracrine factors in mitigating ovarian ageing across various models is systematically summarized in Table 1.

**Table 1:** Summary of experimental studies on MSC-based therapy for ovarian ageing

Induction Model of Ovarian Ageing	Model Characteristics	MSC Type Used	Main Mechanisms and Effects	Reference
Chemotherapy-Induced	Rapid follicle depletion, granulosa cell apoptosis, and DNA damage.	BMSCs	Suppressed chemotherapy-triggered germ cell apoptosis and DNA damage.	[39]
Natural Ageing	Declined follicle quantity and quality.	BMSCs	Regulated mitochondrial function and inhibited apoptosis; no signs of tumors were observed in long-term studies.	[40]
Chemotherapy-Induced	Premature ovarian failure with disrupted hormone levels.	hPD-MSCs	Increased serum estradiol levels, reduced FSH and LH levels, and inhibited granulosa cell apoptosis (via the IRE1α pathway).	[41, 42]
Chemotherapy-Induced	Ovarian failure and follicular atresia.	hAD-MSCs	Multiple mechanisms: Anti-apoptosis, hormonal secretion regulation, angiogenesis promotion, growth factor secretion, and immunomodulation.	[43, 44]
Natural Ageing	Declined oocyte quality and mitochondrial dysfunction.	MenSCs	Upregulated mitochondrial pathway-related genes and improved the follicular status of aged ovaries.	[45]
Natural Ageing	Declined ovarian reserve function.	hUC-MSCs	Paracrine action: Promoted ovarian expression of IGF−1, HGF, and VEGF, improving the local microenvironment.	[15]
Chemotherapy-Induced	Primary ovarian insufficiency, featuring excessive autophagy in granulosa cells.	hUC-MSCs	Involvement of IGF−1: Attenuated excessive autophagy in granulosa cells by activating the Nrf2/HO−1 signaling pathway.	[60]
Chemotherapy-Induced	Ovarian damage and functional decline.	human amniotic-derived MSCs	Paracrine secretion of multiple factors: Secreted FGF2, IGF−1, HGF, and VEGF; regulated apoptosis (decreased Bax, increased Bcl−2); promoted angiogenesis and follicular development.	[62]

### The important role of IGF-1 in ovarian physiology and ageing

Studies on the effects of IGF-1 on related cells in the ovary revealed that IGF-1 is present at every stage of oocyte development, suggesting that it may be directly involved in the development and maturation of oocytes [63]. Other studies have also indicated that by acting on GCs and oocytes, IGF-1 can regulate cell proliferation, differentiation, survival, steroidogenesis, and oocyte maturation [64]. The mRNA expression level of IGF-1 was correlated with the growth rate of secondary follicles [65]. IGF-1 can also promote the activation and proliferation of primordial follicles through the PI3K/AKT pathway [66]. It may improve oocyte quality, thereby promoting early embryonic development and blastocyst formation [67, 68]. IGF-1 and its binding protein system interact with the renin-angiotensin system during follicular growth and ovulation, enhancing ovarian angiotensin II production and follicular development by stimulating intrafollicular plasminogen activator activity [69]. These studies demonstrate that IGF-1 plays a key role in the development, maturation, and functional regulation of ovarian cells and influences follicular growth and ovulation through multiple pathways.

### Other paracrine factors and pathways in MSC-mediated ovarian regeneration

It should be noted that the therapeutic efficacy of MSCs is not solely attributed to IGF-1, but rather results from the synergistic effects of a complex paracrine cocktail. Other key factors secreted by MSCs include: VEGF, crucial for reconstructing blood flow in ovaries [70]; HGF, which promotes cell survival and motility; and FGF2, supporting follicular development and angiogenesis [71, 72]. Additionally, exosomal therapy has emerged as a promising approach for improving ovarian function and preserving fertility in chemotherapy-induced POI mouse models [73]. Exosomes carry a variety of bioactive molecules [74] and can modulate the local ovarian microenvironment in POI rats by influencing signaling pathways related to immunoregulation, cell viability, inflammation, fibrosis, and metabolism [75]. However, the precise mechanisms underlying these effects require further investigation.These factors, working synergistically with IGF-1, collectively contribute to the restoration of ovarian microenvironment and functional recovery.

## Molecular mechanism through which IGF-1 improves ovarian ageing

### Biological properties of IGF-1

IGF-1 is a peptide growth factor named for its structural similarity to proinsulin [76]. It is produced primarily in the liver through the endocrine action of growth hormones. Upon stimulation by growth hormones, IGF-1 is secreted into the bloodstream and exerts its systemic effects by acting on target tissues via the circulatory system, thereby mediating the overall functions of growth hormones. The circulating level of IGF-1 is regulated through a negative feedback mechanism [77]. Studies have shown that IGF-1 is synthesized not only in the liver but also in other cells, such as smooth muscle cells, endothelial cells, and neurons [78, 79]. Under the regulation of growth hormones and local factors, it acts in a paracrine or autocrine manner on neighbouring cells or on itself, playing a crucial role in local cell growth and repair [80]. In the circulation, IGF-1 exists in both free and bound forms. It primarily reversibly binds to insulin-like growth factor binding protein 3, which regulates the level of free IGF-1 in circulation and thus controls its biological activity. Upon binding to the insulin-like growth factor 1 receptor (IGF-1R), IGF-1 activates downstream signalling pathways to exert its biological effects. It plays important roles in various physiological processes, such as growth and development, cell proliferation and differentiation, wound healing, anti-inflammatory responses, lifespan regulation and ageing, and immune modulation [81–83].

### IGF-1R: gateway to signal transduction

IGF-1R is a transmembrane glycoprotein receptor belonging to the receptor tyrosine kinase family. Its gene is located on the long arm of chromosome 15 and spans more than 100 kb of genomic DNA [84]. Mature receptors have a heterotetrameric structure, consisting of two extracellular α-subunits involved in ligand binding and two transmembrane β-subunits containing tyrosine kinase domains in their cytoplasmic regions [85]. It is widely expressed on the cell surface and functions primarily by binding to IGF-1 and insulin-like growth factor 2. The role of IGF-1R is fundamental to development and survival; impaired IGF-1/IGF-1R signalling during growth and development can lead to growth retardation, malformations, and intellectual disabilities, whereas complete knockout of IGF-1R expression, resulting in the total absence of IGF-1/IGF-1R signalling, directly leads to organism death [86, 87]. IGF-1R is present not only on the cell membrane but also in the nucleus. The nuclear translocation of IGF-1R may occur through various pathways, such as endocytosis and lysosomal transport [88]. These pathways are closely related to the IGF-1-induced ubiquitination of IGF-1R and accelerated degradation and potentially play significant roles in the degradation process of the receptor [89]. Additionally, the activation of IGF-1R can regulate the expression of its own gene. Experiments have confirmed that IGF-1R can stimulate the activity of its homologous promoter by directly binding to the IGF-1R promoter region, thereby regulating its own gene expression at the transcriptional level [90]. Furthermore, IGF-1 can activate the PI3K/AKT/HIF-1α signalling pathway through its receptor, subsequently upregulating HOXA13 expression. Ultimately, HOXA13 binds to the promoter region of the IGF-1R gene and activates its transcription, upregulating IGF-1R expression [91]. The IGF-1/IGF-1R signalling pathway plays a critical role in various stages of life and in multiple organs. The bidirectional regulation of IGF-1R expression by IGF-1 and the maintenance of its dynamic equilibrium are essential for the body to remain in a normal state.

### IGF-1/IGF-1R downstream signalling pathway and cellular effects

**Fig. 2:**
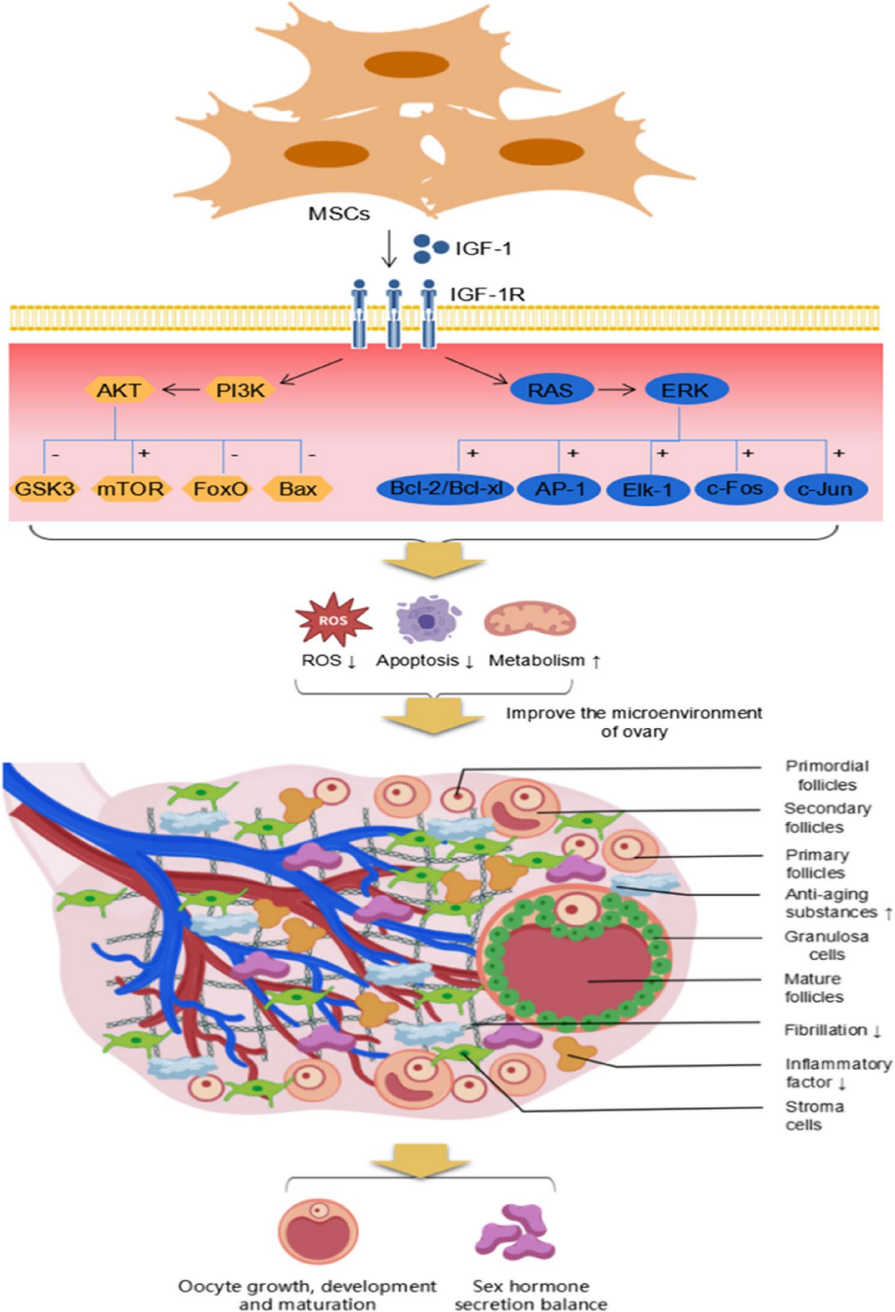
Proposed schematic model of the molecular mechanisms by which MSC-secreted IGF-1 ameliorates ovarian ageing MSCs secrete IGF-1, which binds to IGF-1R on the surface of ovarian cells, activating PI3K/AKT and Ras/ERK-mediated signaling pathways. This inhibits the expression of GSK3, FoxO, and Bax while promoting the expression of mTOR, Elk-1, c-Fos, c-Jun, and AP-1. These molecular changes reduce oxidative stress, inhibit apoptosis, enhance cellular metabolism, and activate autophagy, thereby improving the ovarian tissue microenvironment (reducing ovarian fibrosis, decreasing inflammatory cytokines, and increasing anti-ageing factors). Ultimately, this promotes follicular growth, development, and maturation, and regulates the balance of sex hormone secretion. (+: activation; —: inhibition)(Created with biogdp.com)

IGF-1R is activated upon binding to IGF-1. The activation of the receptor triggers a series of cellular responses, including proliferation and growth, and protects cells from programmed cell death or apoptosis [92]. Among these pathways, the PI3K/Akt pathway is a key signalling pathway through which IGF-1 exerts its effects. After IGF-1 activates its receptor, it activates PI3K via insulin receptor substrates. The product of PI3K activation, phosphatidylinositol 3,4,5-trisphosphate (PIP3), is recruited to Akt [93]. The activation of Akt acts on multiple downstream targets, such as Bcl-2/Bax [94], glycogen synthase kinase 3 (GSK3), mammalian target of rapamycin (mTOR), and FoxO family transcription factors [95–97]. The activation or inhibition of these downstream targets leads to various cellular effects, including proliferation, growth, differentiation [98], inhibition of apoptosis, metabolic regulation [99], immune responses [100], and tissue repair [101, 102]. Another mechanism through which IGF-1 produces biological effects involves the activation of the Ras/Raf/MEK/ERK pathway [103]. After IGF-1 binds to its receptor, it sequentially activates Ras protein, Raf kinase, and MEK, ultimately leading to the activation of ERK through phosphorylation. Once ERK is translocated into the nucleus, it regulates the activity of transcription factors such as Elk-1, c-Fos, and c-Jun, promoting the G1/S phase transition of the cell cycle and playing a critical role in cell cycle progression and proliferation [104]. The activation of the transcription factor AP-1 results in the degradation of the extracellular matrix and enhanced cell migration [105]. It can also act on Bcl-2/Bcl-xl to inhibit apoptosis [106]. Additionally, IGF-1 can regulate cell migration and adhesion through interactions with extracellular matrix receptors such as integrins [107]. In summary, IGF-1 influences cell proliferation, differentiation, metabolism, and apoptosis through multiple pathways. These effects are closely associated with delaying or reversing cellular and tissue ageing. (The regulatory mechanism of IGF-1 is illustrated in Fig. 2.)

## Conclusions

The decline in ovarian function poses a significant challenge to women’s reproductive and overall health. Preclinical evidence strongly suggests that IGF-1 secreted by MSCs serves as an important mediator against ovarian ageing, leveraging its potent effects on cell survival, metabolism, and proliferation through pathways such as PI3K/Akt and MAPK/ERK.However, this promise must be contextualized within the limitations of the current evidence base. The vast majority of supporting data derive from animal models, while direct causal evidence from in vivo loss-of-function studies remains scarce. The transition from promising preclinical findings to viable clinical treatments faces considerable obstacles, including ensuring the safety and precise targeting of transplanted MSCs, understanding the functional heterogeneity of MSCs from different sources, and validating long-term efficacy and safety in large-scale human trials. Future research should prioritize decoding the synergistic network between IGF-1 and other paracrine factors, developing strategies to enhance and control IGF-1 delivery, and conducting rigorous clinical trials. Through these efforts, the potential of targeting the IGF-1 pathway in MSC-based therapies can be fully and safely realized.

## Data Availability

No datasets were generated or analysed during the current study.

## Abbreviations

AKT: Protein kinase B
AMH: Anti-Müllerian hormone
Bax: Bcl-2-associated x
Bcl-2: B-cell lymphoma-2
Bcl-xL: B-cell lymphoma-extra large
BMSCs: Bone marrow mesenchymal stem cells
eNOS-NO: Endothelial nitric oxide synthase/nitric oxide system
ERK: Extracellular regulated protein kinases
FGF2: Fibroblast growth factor 2
FSH: Follicle-stimulating hormone
GCs: Granulosa cells
GSK3: Glycogen synthase kinase 3
hAD-MSCs: Human adipose tissue-derived mesenchymal stem cells
HGF: Hepatocyte growth factor
HIF1α: Hypoxia-inducible factor 1-alpha
HOXA13: Homeobox A13
hPD-MSCs: Human placental-derived mesenchymal stem cells
hUC-MSCs: Human umbilical cord mesenchymal stem cells
IGF-1: Insulin-like growth factor 1
IGF-1R: Insulin-like growth factor 1 receptor
IGF-2: Insulin-like growth factor 2
IGFBP: Insulin-like growth factor-binding protein
IL-1: Interleukin-1
IL-6: Interleukin-6
LH: Luteinizing hormone
MEK: Mitogen-activated protein kinase kinase
MenSCs: Menstrual blood-derived mesenchymal stem cells
mTOR: Mammalian target of rapamycin
MSCs: Mesenchymal stem cells
PI3K: Phosphatidylinositol 3-kinase
PIP3: Phosphatidylinositol (3,4,5)-trisphosphate
POI: Primary ovarian insufficiency
Raf: Rapidly accelerated fibrosarcoma
Ras: Rat sarcoma virus
ROS: Reactive oxygen species
TNF-α: Tumor necrosis factor-alpha
VEGF: Vascular endothelial growth facto
